# Dating Apps and Shifting Sexual Subjectivities of Men Seeking Men Online

**DOI:** 10.1007/s12119-024-10231-1

**Published:** 2024-04-25

**Authors:** Barry D Adam, David J Brennan, Adam WJ Davies, David Collict

**Affiliations:** 1 Department of Sociology and Criminology, University of Windsor, Windsor, Ontario N9B 3P4 Canada; 2 Factor‑Inwentash Faculty of Social Work, University of Toronto, 246 Bloor St. W, Toronto, Ontario M5S 1V4 Canada; 3 Department of Family Relations & Applied Nutrition, University of Guelph, Guelph, Ontario N1G 2W1 Canada; 4 Ontario Institute for Studies in Education, University of Toronto, 252 Bloor Street West, Toronto, Ontario N1G 2W1 Canada

**Keywords:** Dating apps, Geosocial networking mobile apps, Gay men, Sexual fields

## Abstract

Leading theories of the recent history of sexuality have pointed to trends toward detraditionalization and precarity in intimate relations, but also to democratization and innovation. This study grounded in 79 qualitative interviews with men seeking men online considers their experiences in light of these theories. The rise of dating apps has generated sexual fields that have shaped the sexual subjectivities of the current era in multiple ways. The narratives of study participants show much more than the hook-up culture that dating apps are best known for. They speak to experiences of superficiality, unmet expectations, and sometimes bruising intersections with hierarchies defined by age, race, body type, gender expression, and serostatus. Yet at the same time, they show a strong aspiration to sociability, social network building, and reach for a language of affiliation beyond the kin and friendship terms of the larger society. Generational comparisons indicate the shifting sexual subjectivities that dating apps have shaped by constituting virtual sexual fields.

## Introduction

Socio-historical studies of sexuality and intimacy have posited two broad visions of the change in western, industrial societies over the last century and a half. One vision, perhaps best articulated in the work of Giddens (1992), Ulrich Beck and Elisabeth BeckGernsheim (1995), and Bauman (2003), constructs contemporary sexuality and intimacy as cast adrift from familiar signposts, where individuals are left to their own devices to create often fragile relationships amidst declining support from tradition, religion, or community (Adam, 2020). Giddens postulated the “pure relationship” as an emergent form in the current era, where people enter sexual and intimate relationships in a voluntary and consensual manner but, lacking the economic and social obligations of earlier epochs, make relationships that last only as long as they are gratifying to both partners. Woltersdorff (2011, p. 165) summarizes these changes as “first, the disappearance of the old idea of perversion in a deregulated sexual market, second, the decline of tradition and the rise of precarity within gender relations, [and] third, the implementation of a contractual ethics of negotiation.” For LGBTQ + people, as well as heterosexuals, this often means facing both freedom and challenges in seeking new relationships in social worlds marked by individualism, competitiveness, and sexual consumerism (Adam, 2016).

The migration of dating to online spaces has perhaps exacerbated these tendencies resulting in what Illouz (2007) calls “cold intimacies,” where those seeking partners find themselves obliged to package themselves for a sexual marketplace, resembling online shopping (Bauman, 2003). Applied specifically to the sexual fields of gay, bisexual, and other men who have sex with men (GBM), the question arises whether the current dominance of dating apps has contributed to a privatization and individuation of LGBTQ + communities, as the urban spaces of the late twentieth century have entered into decline, disappearing beneath gentrifying trends in major cities (Ghaziani, 2014; Miles, 2017).

An alternative vision of current forms of sexuality and intimacy celebrates these changes as part of a “long process of the democratization of everyday life” (Weeks, 2007), where sexual and intimate arrangements have devolved from church and state into the hands of individuals who, in turn, have created new forms of loving and living together, including a flourishing of same-sex relationships (Adam, 2006; Stacey, 1990; Weeks et al., 2001). For GBM in particular, dating apps (sometimes referred to as geosocial networking mobile apps in the research literature), allow a largely hidden population to become visible to each other on a virtual plane and potentially to find one another, escaping, if momentarily, from regulated social environments. They may be particularly useful for rural people and migrants, who otherwise have few options for meeting people or for locating themselves in new communities (Lennes, 2021; Miles, 2018; Wu & Ward, 2018).

Dating apps may, then, be construed as sexual fields (Green, 2014; Regan, 2021) that “have not only shaped queer male spaces over the past three decades, but increasingly constitute what these spaces are, how they are performed, and who is able to access them” (Miles, 2018, p. 2). The shape of these fields has begun to emerge through recent research. A survey of Grindr users in the southern United States on their motivations for app use found that “over one-third of the men reported using these apps to meet other men for sexual encounters (38.0%), and the second most common reason was using these apps to ‘kill time’ when bored (18.5%), followed by using these apps to make friends with other men (17.4%), to find a boyfriend or romantic partner (14.1%), and to meet other gay and bisexual men to date (10.9%)” (Goedel & Duncan, 2015, p. 5). Interviews with GBM on Chinese dating apps revealed a strong interest in pursuing human connection and sociability, such as “the exhilaration of a casual conversation with interesting people” along with “sexual encounters in which they could feel connection and intimacy” (Wu & Ward, 2020, pp. 348–349).

While apps may facilitate sexual and romantic intentions, at the same time they appear to generate sexual fields that place several impediments to their realization. The format of the app profile typically provides very limited information about potential partners, removes them from their social context, and manufactures a virtual identity with an uncertain relationship to the person it purports to represent (Blackwell et al., 2014, p. 1128). For many users, then, the experience of looking for human connection is characterized by rapidity, instrumentality, and superficiality where meaningful or durable relationships seem hard to find (Licoppe, 2020; Wu & Ward, 2020) and the apps appear at times to facilitate deception, catfishing, and rejection (Lauckner et al., 2019). Racialized people, in particular, find that the cloak of anonymity provided by apps makes expressions of overt racism even easier to make than in face-to-face interactions (Hammack et al., 2021) and others feel the sting of rejection because of their body type, gender expression, age, and other characteristics. It is perhaps not surprising, then, that a “poor ratio of online conversation to in-person meet-ups” is commonly reported (Miles, 2017, p. 1604) and one study found half of app users to be “discouraged users” who perceived apps to be unreliable in helping to find dates or hookups (Choi & Bauermeister, 2022).

This study, then, accesses qualitative interviews with GBM concerning their experiences with apps, in order to better understand the structure of sexual fields generated by the apps which shape their expectations and experiences for intimacy and sex. Set within a larger socio-historical context, these interviews also provide cues to how this structuration moves over time, as older and younger men offer somewhat varying accounts of these shaping processes.

## Methodology

Participants for this study were drawn from a larger survey-based study (Brennan et al., 2022) of the views of GBM on online outreach by AIDS service organizations and public health agencies to address sexual health questions (*N* = 910). We sought to gain a better understanding of the virtual environment that structures outreach work by exploring how GBM navigate questions of health and safety while interacting online, with specific questions asked regarding the experiences of app users navigating online social and sexual spaces. Eligibility for study participation included identifying as male (cisgender or transgender), reporting sex with a man in the previous year, being sexually or romantically attracted to other men, or identifying as gay, bisexual, queer, or Two-Spirit. Recruitment was carried out through social media sites, dating websites and apps, and through listservs of local community-based organizations. Data collection was completed online with a baseline and follow-up questionnaire, that included a question asking study participants if they would be willing to be contacted for a later interview on their online experiences. Participants in the qualitative study were recruited from the larger study with an eye to maximizing diversity as defined by age, ethno-racial identification, cis or trans identity, urban/rural residence, and HIV status. Interviews used a semi-structured guide with scripted probes aimed at providing an in-depth exploration of participants’ experiences of dating apps, including motivations for use, comparisons with meeting men offline, overall satisfaction, different or unfair treatment while using apps, as well as contact with online health outreach workers. Interviews took place in person in a university office or over the phone. Participants were offered $30 CAD for their time. Research Ethics Boards of the University of Toronto, University of Windsor, University of Victoria, and Toronto Metropolitan University reviewed the research studies before recruitment began.

The interviews were audio recorded, transcribed, and analyzed in NVivo 12 using a constant comparative approach to identify emergent themes. The full transcripts were first coded line-by-line for all possible themes by the first author, then discussed with the co-authors to identify salient themes and excerpts indicative of the range of views (Smith et al., 2009). Themes were then organized to create a coherent, analytical narrative. All data were included in the analysis, including questions regarding experiences navigating social and sexual online spaces and participants’ opinions of and experiences with online sexual health outreach.

## Findings

The final purposive sample consisted of 79 participants whose ages ranged from 16 to 77. The mean age was 32 and the modal age group was 18–29 (*n* = 35). Ethno-racial identification was White (*n* = 29), South or East Asian (*n* = 16), African/Caribbean/Black (*n* = 10), Latino (*n* = 7), Indigenous (*n* = 12), and other (*n* = 5). Fifty-one (65%) identified as gay, 10 (13%) as bisexual, and the rest with a range of categories such as queer (*n* = 11, 14%), pansexual (*n* = 5, 6%), asexual (*n* = 4, 5%), or questioning, mostly straight, or two spirit (*n* = 8, 10%). Nine (11%) were HIV-positive. Eight identified as trans men. The modal educational level was a university degree (*n* = 40), 26 had some postsecondary education, and 13 had high school or less. Most (*n* = 50) reported incomes under $40,000 CAD per year, 15 earned between $40,000 and $69,999, and 14, $80,000 or more. Twenty-one (32%) were immigrants to Canada. Eleven (14%) reported rural postal codes. Index numbers of individuals quoted in the following text link to demographic characteristics provided in the Appendix.

The findings reported here are based primarily on experiences with the three most frequently named dating apps, Grindr, Scruff, and Tinder, and occasionally with other apps or dating websites. There were widely shared perceptions that Grindr is the most popular dating app, appealing to a younger demographic with primarily sexual intentions, while Scruff participants were viewed as somewhat older and more likely to favour more lasting connections, and Tinder was thought to be more oriented to relationship development. At the same time, many study participants remarked on the overlap of membership among these apps and participants’ narratives show a good deal of diversity in practice and perception.

### App Affordances: Human Connection

Interview narratives show just how variable app use is. The men in this study mention options that they valued in app use that were not easily available to them in face-to-face interactions, such as opening the door to simple affirmations of being seen or being affirmed as desirable. Their ease of use reduced the anxiety of approaching someone new and certain kinds of openness had become increasingly normative online, particularly in sharing sexually related preferences and images. Several men valued app communication for both giving and receiving information in advance of meeting in order to screen prospective partners.

A major theme running through interview narratives was the value of simple human connection and sociability afforded by app use, meaning that participants from various regions, whether urban, rural, or otherwise, reported the importance of human connections and relating with others online. Though perhaps best known for their potential to generate hook-ups, a large proportion of the narratives treated the satisfaction of feeling socially connected and overcoming loneliness as primary considerations. While some expressed sociability as their primary interest in app use, others arrived at a realization that much of what took them back to checking app activity was the pleasure of conversation, even after the potential for an encounter had passed or never really existed. This app user characterized his anticipation this way: “it’s like what kind of crazy chat adventures am I going to encounter today? It’s just the general sort of smorgasbord of interesting chats” (55)^1^ that kept him engaged. Another concurred that “I enjoy just talking to random people that I would have, probably, no reason to run into in general life” (17). Several reported engaging in chats that extend to other countries as “some of them are halfway around the world and I’ll never meet them, but it’s like, that was nice–thanks for spending two hours chatting–that my evening suddenly wasn’t so boring” (36).

For men in rural areas with little or no overt gay geography, “going online was just the only way I could think of to even have a chance at meeting another gay man. Simple as that” (57). And for immigrants, apps provided some ability to keep in touch with expatriates and also to rebuild contacts in a new society as in “trying to meet friendly people, connection–maybe the type of the connection that I used to have back home in Africa, which I think is very rare here, but I still look for it” (39). A few had overtly nonsexual interests, saying “I mean most of them they want sex, but I don’t want sex. I just want someone to talk to” (32). One interviewee had some success in pursuing a specifically asexual interest in cuddling: “I think before, people expected the cuddling to come with the sex, but now they’re asking for cuddling by itself, as its own thing, because they realize that they need and want that, and can get it” (48).

### Sexual Connection and More

Not surprisingly, explicitly sexual interests also recur as a major theme in narratives about app use. Some have a singular focus on apps as a means to sexual connection and found lengthy chat to be an unfortunate distraction—“I’ve never really needed friends; ultimately, my main goal was just to find a hook-up” (24)—while others wanted to chat a lot to find the rapport that can make a sexual encounter more satisfying:

I usually chat with them for a pretty long time, a couple of weeks, sometimes a month before I even meet them up, so I think there is some kind of a rapport that you have already created with them, some kind of connection between both of you, before you actually meet. (43)

While for many, “sex is the top of the list, for sure” (51), sexual encounters open the way for the development of “friends with benefits” (06) or more. A common narrative was being open for the possibility of a relationship:

Really, I’m looking for a relationship, but you’ve got to meet them first, so usually it ends up being a hook-up first, and then seeing if it goes on to something else from there. (04)

Some characterized their participation on the apps specifically as “husband shopping” (15) or looking for “a boyfriend and hopefully marriage one day” (37).

A few observed that dating apps have opened additional avenues for the expression of queer desire both among gay and nongay identified men. One man found that despite being, in his own words, “an older, fat, hairy guy” (23), that there is “a shocking amount of guys” who find him interesting. He speculates that “there’s a kind of a social regulation going on in the clubs–certain people can approach certain people and they can’t approach other people–whereas with the apps, you can try it out in a way that it’s lower commitment and it’s a bit more private.” Whether it is an interest in kink, fetish, older or younger partners, or body types that are not idealized in public media, some found online spaces to be more conducive to one-to-one communication of minority or adventurous preferences. The low barrier virtual environment, where participation may be as easy as a click on a cellphone screen, also allowed a wider range of men with homoerotic interests to explore their feelings. While some expressed surprise or even dismay at the number of men online who are apparently married to women or identify themselves as “straight,” this respondent observed that new opportunities for sexual self-discovery have come about as “we’re in an environment now where the rigidity, the policing of masculinity… has loosened up” (23).

### Building Networks

App use, then, raises larger, longstanding questions about how apps contribute to the ways in which LGBTQ + people build families and communities. While creating families consisting of same-sex parents and their children has occupied a great deal of scholarly and media attention in recent years, the great majority of GBM piece together networks and “chosen families” in ways that have few public models or institutional supports. Being new in town, looking for gay spaces, feeling displaced, recently out of a relationship, or just lonely, all figure in the impetus to make new connections, both sexual and friendly. Suburban and rural men, as well as immigrants from other countries all aim to find a place for themselves among supportive social networks. For this recent immigrant, “Grindr was an opportunity to meet people for sex for sure and also, I can meet people to make friends or improve my English” (25). The exigencies of the job market often meant that people move considerable distances to pursue new opportunities:

I recently moved back to the city, so just sort of meeting people and stuff, because it’s really difficult when you’re gone for a while, and then you come back and you’re like, oh yeah, half of my friends are gone. I don’t know anyone. (20)

Similarly, schooling can result in relocation: “it was nice when I was studying abroad because it was a way to just connect to people in a city where I knew literally nobody. I always met interesting people that way” (18).

Several narratives dealt specifically with being already connected in nongay social networks but wanting to find like-minded companions: “I don’t really have any gay friends, per se, just a lot of girlfriends, so I don’t have anyone to talk about that with” (12). A common experience, recurring through the interview narratives, was that sexual encounters led to building a circle of (nonsexual) friends over time: “I’ve been on and off on these apps for eight to nine years and made quite a lot of friends actually” (19). One man described himself as “in a transitional phase where I’m moving more into, ‘okay, so, it’s less about being horny and more about finding a tribe or a group of folks’” (79). Another who described his app use as “a sexual outlet,” quickly acknowledged having “met people from all around the world” and found many of these online friends became his primary social network when “in 2015, I was, out of nowhere, hit with a diagnosis of cancer…and I found most of my support from strangers online” (15).

### Building Networks while Already Partnered

Ten members of the sample participating in this study reported in their interviews being in a committed relationship. Some single men looked askance at app users in relationships remarking, “It gets frustrating for a person who is actually looking for a partner, because you keep finding guys who are just like, ‘I’m married, I’m in a relationship’” (35) while another noted with a certain puzzlement that “what’s weird on Tinder is that a lot of couples use it, is what I’ve found. People make joint profiles, like Matt and Tom; it’s like two men seeking a third” (33). Narratives of virtually all of these men did not see their primary relationship as excluding the wide range of sexual and human connections or network building that interested unattached men. Some asserted a boundary between their primary relationship and additional sexual pursuits–“I’m in an open relationship, so it’s mostly I’m looking for sex” (49)—but more expressed interest in “some kind of friendly relationships” (23), “connections” (39), “an ongoing thing where it has undertones of a friendship” (40), “people to go out and do stuff with” (59), and “someone [who] is friendly and makes good conversation” (70). These phrases suggest a reach for a language of affiliation to describe relationships that do not fit the kin and friendship terms of heterosexual society. Having a primary relationship does not necessarily obviate a desire for larger networks of support, friendship, playful relations, or sex buddies. One man, having faced online criticism from time to time for his presence on an app, countered, “I have a partner and we have an open relationship. We’re very clear about that, but they’re like, ‘Oh, is that why you’re on there? Just to hook up?’ ‘Well, no, I’m meeting people,’ because…. there’s a whole spectrum of relationships out there in terms of, you just want friendship.” (36)

### Apps as Sexual Fields

The contours of the sexual fields opened by the apps offer a range of opportunities and possibilities, but this virtual geography comes with constraints and disappointments. Hopes and aspirations come up against frequent missed connections and outright rejection. Several study participants remarked that app structures encouraged superficiality, unmet expectations, and even deceptive practices. Interaction with images on screens in a context of relative anonymity allowed for a masking of the humanity of the individuals behind the profiles and the illusion of instant availability diminished the likelihood of participants taking the time to engage in any depth with other people. One man found, “there’s something about the instant nature and instant satisfaction that I think leads people to not treat each other as humans…. Here’s a wall of people, choose one, use them, throw them away–like the expendability of people, as opposed to actual genuine connections.” (18)

The promise of easy availability left several individuals feeling, “you’re a filler until something else catches my eye or something shiny catches my eye, so I found people don’t treat people like people” (15) and another concluded, “If you’re not a 9 or a 10, don’t even try, people don’t care. Because the ocean is so big, catching a fish is like–you don’t like it? You can throw it back and catch a new one” (67).

Misleading or deceptive presentations of self on apps were a common experience. “With the online interactions, you don’t know what you’re getting at the other end and I’ve also been catfished through a couple of apps, and that was very disappointing” (17). This individual reflected on how his own hopes set himself up for disappointment even if the prospective partner had not intended to deceive:

I’ll be on someone’s profile, I’ll be looking through them. They have really cool photos and I see like maybe they have a dog and I’m just like–I paint this picture in my head of this person’s life … projecting what I’d like them to be like. So you obviously paint a perfect picture and you’re almost always like a little disappointed, but I’m doing it to myself. (63)

Online interactions were seen as tenuous: “it’s hit and miss. Usually, I would say… I end up needing to engage in ten conversations; I may meet one maybe. Actually, it’s probably lower than that” (46). Several men remark on the silences and disappearances that are frequent outcomes of online chat: “I was chatting with one guy and we’d exchanged photos and stuff, and he seemed to be totally interested, and we’d agreed to meet up. I said, ‘okay, I’ll head out now. What’s your address?’ Then he just went silent…. They just disappear” (23). And finally, more than one interviewee did meet up and then went through with sexual encounters despite misgivings: “and then they come over and it’s like, oh god, what did I just get myself into? I’m remembering one of them and it’s like, I just wanted it to end” (43) and another said, “I was really disappointed, but I was so horny, I just kind of went through the encounter” (29).

### Intersections with Age, Race, Body Type, Gender Expression, and Serostatus

The apps appear to amplify or at least, facilitate the enactment of discriminatory practices based on age, race, body type, gender expression, and serostatus. Patterns of rejection appear to reinforce the desirability of the archetypal young, white, fit, masculine body type. The great many individuals who do not embody this standard felt the sting of overt or covert rejection in several instances. A Black interviewee found, “people are bigoted on Grindr, like they’ll say whatever like racial slur or whatever is coming to mind, versus in person someone might not say that to you” (29). Another man said, “I’m half Asian, so I find a lot of times, when I’m trying to interact with somebody, I would just get flat out, ‘I don’t like Asian people.’… It is very racist. But people have their preferences, I understand that, but it hurts” (17). Sometimes racism could be more covert: “They don’t say, ‘oh, I don’t like you because you’re Asian.’ They never say that. No, no. I don’t ever experience that, but I feel … either it’s because of my race or because of my face” (03). And another found, “Say a guy is interested, because obviously, for the most part, I’m white passing, but when I do indicate that I am mixed [Asian], some guys just get hostile, or just ignorant, or whatever” (64). In some instances, racial stereotypes may be sexualized. This Latino interviewee found that “sometimes you hook up, and then they make comments about my ethnicity that is kind of racist and off-putting” (48). Racialized men are left wondering how other gay men can so easily make them feel unwelcome as community members and as citizens. “It’s still a little bit surprising because you think being gay–we’re discriminated against for something we can’t change–that we would be automatically more understanding of other things that people can’t change” (50). And again, “In Canada, I thought that people would be very inclusive, kind of in the way they speak as well, but I don’t think it happens on Grindr” (39).

In a community with a wide range of gender expression, study participants remark on the pressure to conform to gender binaries. One respondent was valued for his ability to embody femininity but was left wondering about the disjuncture between image and self:

I also dress up like a drag queen, or whatever you want to call it, and so my female persona, she picks up wherever she goes…. She can be on Tinder and it’s like guys are so crazy, it’s ridiculous…. I guess I’m just a very beautiful woman. I do see that and that sometimes gets me a little bit bothered, because I’m just like, I’m the same person. People would rather just sort of fall in love with this image—‘oh my god, you are so beautiful’--as opposed to, hey, you realize that I have so much to offer. (35)

Others talk about monitoring themselves so as not to be perceived as “flamboyant” (32) or striving to present as sufficiently masculine: “I’m pretty feminine; I have more so feminine features and a lot of the guys in my city are more interested, a lot of the times, in the masculine types, so there’s been a few times where people have made kind of just negative comments on my appearance” (24). Another discussed the expectations regarding Black masculinity: “I’m black and I’m the top and they’re expecting me to be super hard-core masculine….[but] I have had enough positive experiences to know that I don’t need to be super masc to be sexy” (09).

Trans men report mixed reactions but often found themselves having to educate others about who they are.

I had some people that would reply saying that they don’t date trans people or that they’re not into people with my junk without even actually asking…. Then other people were saying, ‘oh, well I’m gay, I’m into men,’ and then essentially just implying ‘well, you’re not a guy,’ and that was no. That was awkward” (14).

Another found men online to be “really chill about it” and says, “I haven’t really had anybody who was transphobic” (08) but incomprehension exacted a toll at times.

I have had to explain to people quite often that you can be a trans guy and then they’re like, ‘oh wow I had absolutely no clue.’ And if they meet me in person, they will have a lot of questions…. Oftentimes I’ll talk to a friend who says, ‘yeah you were the first trans guy I ever met but now I know this guy and this guy and this guy.’… They get more comfortable. Over the span of a few months, they unwind a little bit. It also helps that I have a sense of humour about me being trans; they feel comfortable around me. (65)

Body type also can be a source of rejection for both those perceived as overweight and those perceived as underweight. Some slightly built men had to cope with the “twink” label and several objected to having their bodies read as indicators of particular sexual preferences or personalities.

I personally don’t identify with the group of twink. I hate that. But I also know that I hit that profile so, more often than not, people will think of me as that and I really hate that I get profiled like that because I think there is more to me than just that I’m a skinny white boy. (45)

Another describes persistently receiving a cold shoulder from men online as “kind of soul crushing…. Because I am smaller, I can’t grow facial hair to save my life, and I am somewhat boyish in appearance, I have a lot of guys who right off the bat will dismiss me as femme, which is a dirty word.” (40)

Age, too, is a characteristic that can be used to screen and reject prospective partners, sometimes in surprising ways. Several young men in this study expressed an exclusive interest in age peers and many older men felt increasingly sidelined from app interaction as they aged. At the same time, some young men in their late teens or twenties found they are dismissed as too young and both older and younger men express ambivalence about the daddy/boy language circulating in gay communities that conjures role expectations based on age. One man in his twenties complained about “when you’re younger and you’re looking for older individuals and they just try to play the ‘oh, well, you’re my kids’ age’ card” (10). Another found “if I’m seeing or chatting with guys that are significantly older than me, then they often will call me boy and daddy’s boy, and I feel a little gross by it” (45). Older men sometimes feared being cast in the role of “sugar daddy” when young men approached them online (31).

Two older men had the experience of being rejected for still being “too young.” One interviewee in his thirties reported chatting with an eighteen-year-old who found him too young—“He wanted somebody, I don’t know, over 50 years old” (43). And even one man over fifty had had “one guy who messaged and he said I wasn’t old enough. I thought … honest to God, it was the first time somebody in my life had said I was too young” (59). One individual found contact with younger men through apps had changed his perception of age-differentiated relationships:

I was 54 years old and I had somebody who was crazy attractive after me and after me and after me, who was, I believe at the time was 25 …. And this 25-year-old talked me into meeting and I found him to be fascinating, brilliant, and not at all the twinkie child that I had imagined.… Many people think that way with age and I was guilty of it on the opposite spectrum, at the opposite end. So, yeah, I have learned. I have to say that the apps are a good education tool sometimes. (15)

Rejection by age, race, body type, gender expression, or serostatus exists within a larger realm of fleeting and often unrealized connections that was widespread among study participants. Rejection by ascribed characteristic, then, often overlapped with a general sense of tenuousness, superficiality, and lack of empathy in online interactions. Racialized men, particularly, were left unsure in diagnosing online behaviour. One Latino participant remarked, “no one has ever said anything right to my face about I don’t want to talk to you because of your ethnic background or because of your looks. They just stop talking so it’s kind of hard to tell what made them stop talking, so I don’t know” (52). And a Black participant observed, “I’ve sent messages to persons and there’s no response and sometimes you feel a little thing like, okay, I guess it may be race, it may be physical fitness, it may be looks, it may be countless of things” (53).

Some study participants talked about developing coping strategies and defenses against the slights and disappointments that seemed almost ubiquitous. Some trans, HIV-positive, and racialized men stressed their statuses on their online profiles so that “people who don’t like that will just stay away, hopefully” (21). Others tried to develop a thick skin or mitigate the pain of psychic wounds received online:

I’ve definitely had a day brought down a great deal just because of a single message and … I really need to let this go. It’s just an idiot. Ignore it, but my entire mood has suffered. (40)

Similarly, “I was always very feminine as a kid…. I am the way I am and I’m not going to change it for some form of app. It’s definitely shitty, but what can I do? Everyone has their own taste. I just wish some people could, you know, keep their mouth shut if they have nothing nice to say.” (24)

A few show some awareness of their own role in issuing rejection and “try to stay away from shaming people, whether it be for their race or their body or whatever, because you don’t want to be like that” (35).

Do not indent here One Black respondent viewed others who make racist comments with pity, saying, Indented quote begins here I actually found myself in a place of compassion because I was like, it must be really rough for him, that here he has these clearly racist views of Black men, and he was reaching out to a Black man to have sex with. Clearly, he seems to have been rejected by everybody else…. I’m like, you poor soul (53).

And another Black respondent talked about how taking sociology courses gave him analytic distance from hurtful comments, noting that.

I still have that kind of thought process of like how this person came to believe all these things, the fact that they’re saying this to me right now….so if they come and do something, say something negative to me, it’s like okay, it’s not my problem (77)

Despite the psychological bruises experienced by many study participants online, there was also an undercurrent of optimism running through various narratives. Several celebrated recent moves made by app organizers to quell racist commentary and felt that there had been a decrease in overtly exclusionary comment directed at racialized people over time. Some mentioned that they had availed themselves of the feedback options that allowed them to report racist messages to app administrators. Others felt that a change had occurred over the last decade with fewer profiles now indicating racial preferences than before. Several narrators who identify as white commented on the white privilege that shielded them from discrimination and expressed empathy with racialized friends who had experienced hurtful comments. Some white men even mentioned that they refused to engage with “guys who have ‘white guys only’ [on their profiles]; I don’t talk to them” (51), seeing an expression of racism as an indicator of bad character among other white men.

HIV-positive men, as well, expressed optimism about an improving social climate as the “prevalence and the information that PrEP has brought about has done a great deal of education for the community around the safety and general health of what it means to be HIV positive and how willing people are to engage sexually with positive individuals” (68).

Finally, of the 35% of study participants who indicated a bisexual or other not explicitly gay identity, none mentioned having experienced discrimination because of their sexuality.

### Generational and Developmental Experiences

The shifting sexual subjectivities of GBM navigating the sexual fields structured by dating apps are perhaps most evident in comparing the narratives of older and younger men. Most of the older men had lived through the transition to the virtual world of sex and intimacy and had a sense of gains and losses in the diminution of a gay world of bars and baths associated with the rise of the apps. Younger men entered into a sexual world defined by apps as digital natives and sometimes experienced offline gay spaces as uncomfortable or unreadable. Several believed they were inept at reading social cues in bars.

[If] somebody came up to me, ‘oh, hi, I think you’re attractive,’ I would probably be really awkward and I’d probably just take it as some random person trying to give me a compliment, not somebody who wants to hook up with me or wants to maybe date me…. But if it was online and if the structure was there of it being like, okay, people are on this site sometimes specifically to look for other people, then I would be more open to seeing it as somebody trying to come on to me or whatever. (21)

Another man in his twenties shared this view:

I have never had that–just meeting someone in person. I’ve gone out, but I’ve never just met with someone in person and hit it off or whatever…. I feel like meeting someone in person, you wouldn’t really know anything about them, as opposed to online, at least you have some sort of bio to read about them. (12)

A respondent in his thirties felt he understood the sexual field defined by apps, but not by bars or coffee shops.

It’s easier to understand what I want [online] because I have listed what I am looking for whereas in person, you’re sussing someone out. Is this a friends thing or are you hitting on me right now? I don’t know. (65)

Another in his twenties summed it up, saying, “I mean, it’s better than the old way because I don’t know how people meet people face to face anymore” (67). The sense that there is no other option was captured by this individual in his thirties who averred, “I hate app dating. It’s so exhausting and it’s so fleeting but on the other hand, it has also been the only way I have really ever met guys” (45).

App participation meshed with coming out among young men, who experienced a new world of sexual opportunity and affirmation. Over time, many talked about a struggle to define a larger agenda of building social networks, friendships, and community beyond “quickies” or they already had a partner but still feel under-networked in their dyads.

## Discussion

Each of the major visions of the social history of intimacy in the west captures a face of the sexual fields created by dating apps. Many of the narratives of study participants give credence to a view of the sexual subjectivities of our era where relatively isolated individuals reach out to one another through the media of dating apps, but they are largely left to their own devices to create often fragile relationships with limited support from tradition, religion, or community. In terms of the larger sociology of intimacy, the modal experience appears to be one of trying to generate “connection” or “chemistry” without many environing social networks, micro cultures, or societal institutions that have traditionally fostered connection at least among heterosexual men and women. A common theme among these study participants is that app structure creates a sense that “a never-ending ‘standing reserve’ of potential partners through which one can swipe offer[ing] the feeling that the options for hookups–shallow relationships that offer short-term emotional satisfaction–is always present” (Paska, 2020, p. 2551). This market-like field exacerbates an apprehension that the default connection with others will be transitory and superficial, and that more durable relationships require working against the grain of the sexual field. Some felt resigned to the dominance of the virtual realm over everyday life and thus over the pursuit of sex and intimacy as well, saying, “most of our lives are online anyway. We don’t have much to talk about in person anymore, especially with things like Facebook or Twitter or Instagram, you basically post a play-by-play of your morning and your day and it’s like, well if I hang out with you now, I’m not going to get to ask you anything because I already know what you’ve done, so we’re just going to sit on our phones anyway.” (08)

App structure with its delimited information, commodification, self-marketing, and game structure further encouraged rapidity and superficial interaction. One man, who stated he had attention deficit hyperactivity disorder, viewed the apps as congruent with his neurological orientation, remarking that “I use the app a lot and it fits with the ADHD a lot, just because there’s an immediacy to it that I actually really like and I take advantage of” (23).

At the same time, the desire for human connection, whether momentary sexual encounters, friends, lovers, partners, or spouses, remains strong. While gay dating apps often have the reputation of being simply for hook-ups, closer examination of their use shows a larger desire for sociability of various kinds (Filice et al., 2021). Their use by men in primary relationships, as well as single men, suggests that the apps offer the promise of providing the means for creating more extended families of choice (Weston, 1991) and might be viewed as potential tools in constructing LGBTQ + forms of support and kinship. Research on the relationships of GBM shows that these relationships very often do, in fact, cross social boundaries defined by age, race, gender expression, and serostatus. Despite the presence of online racism, for example, interracial relationships have been found to be more common about GBM than among heterosexual couples (Jepsen & Jepsen, 2002; Lundquist & Lin, 2015).

## Conclusions

Since the interviews for this study were conducted as part of an exploration of the ways GBM navigate questions of health and safety while interacting online in the context of outreach work done by AIDS service organizations, there are unanticipated themes reported here that point toward a need for further research that could not be fully pursued here. Phillip Hammack and colleagues (2018), for example, have postulated that GBM fall into generations whose experiences have been shaped in a fast-moving historical landscape marked by the impact of HIV/AIDS, major changes in civil rights and liberties, and the virtualization of dating by apps. Comments made by many younger men showed how they feel entirely contained in the sexual fields created by dating apps (Giano, 2019) and appear to have a receding awareness of the bars, baths, and community groups better known to older generations, suggesting that apps have been re-shaping sexual subjectivities in recent decades.

Apps make visible a virtual dimension of men desiring men who are otherwise hard to identify in everyday life. Easy initiation of contact has diminished barriers to expressing queer desire, including among “mostly straight” or nongay identifying men. At the same time, the cloak of anonymity facilitates insensitive treatment of others without the constraints of everyday civility, a sense of ephemeral contact, and quick rejection. These qualities, along with app formats that contain checklists of preferred characteristics, structure sexual fields that remain especially painful for racialized men (Mowlabocus, 2021, p. ch 5), trans men, HIV-positive men, and men whose bodies and gestures do not conform to prevailing standards of masculine presentation. As one study participant of Asian descent observed, “you can make these very quick and large assumptions about a person because you have this profile, this caricature of the person you’re going to meet, and that informs it all and it makes … those biases more apparent” (45). The ostensible abundance of potentially available partners and easy contact seem counterbalanced by a sense of difficulty in developing relationships that are durable and meaningful.

The question remains whether the migration of the search for intimacy to the virtual world facilitates extending social networks, or whether it undermines community by fostering dyadic chains, while it reduces real world space for being together in a larger collectivity. These narratives of GBM seeking connection through app use also show evidence of the generation of a new realm of sociability, valuation of under-appreciated body types and sexual interests, and extended possibilities for romantic and friendship formation, thereby offering support for diverging macro-sociological visions of historical change in sexual subjectivity.

## Appendix

Demographic characteristics of quoted interviewees.

03 Asian, 18–29, HIV-.

04 Asian, 30–49, HIV-.

06 White, 50+, HIV-.

08 Indigenous, 16–17, HIV-, trans.

09 African/Caribbean/Black, 30–49, HIV-.

10 White, 18–29, HIV-, rural.

12 Other ethnicity, 18–29, HIV-.

14 White, 18–29, HIV-, trans.

15 White, 50+, HIV+.

17 Asian, 30–49, HIV+.

18 White, 18–29, HIV-, trans.

19 Asian, 30–49, HIV-, recent immigrant.

20 Latino, 18–29, HIV-.

21 African/Caribbean/Black, 18–29, HIV-, trans.

23 Other ethnicity, 30–49, HIV-.

24 Latino, 30–49, HIV-.

25 Latino/Indigenous, 18–29, HIV-, rural.

29 African/Caribbean/Black, 18–29, HIV-.

31 Indigenous, 30–49, HIV-.

32 African/Caribbean/Black, 18–29, HIV-, recent immigrant.

33, White, 18–29, HIV-, trans.

35 Latino, 30–49, HIV+.

36 Indigenous, 30–49, HIV-.

37 Asian, 18–29, HIV-.

39 Indo-African, 30–49, HIV-, recent immigrant.

40 White, 18–29, HIV-.

43 Asian, 30–49, HIV-.

45 White, 18–29, HIV-.

46 Asian, 30–49, HIV-.

48 Latino, 30–49, HIV-.

49 Asian, 30–49, HIV-.

50 Asian, 18–29, HIV-.

51 White, 18–29, HIV-.

52 Latino, 40–49, HIV-.

53 African/Caribbean/Black, 30–39, HIV+.

55 Asian, 30–49, HIV-.

57 White, 18–29, HIV-.

59 White, 50+, HIV-.

63 White, 18–29, HIV-, rural.

64 Asian, 30–49, HIV-.

65 White, 18–29, HIV-, trans.

67 Indigenous, 18–29, HIV-, rural.

68 White, 30–49, HIV+.

69 Asian, 30–49, HIV-.

70 White, 30–49, HIV-.

77 African/Caribbean/Black, 18–29, HIV-.

79 African/Caribbean/Black, 18–29, HIV-.

## Footnotes

1 Socio-demographic characteristics of study participants may be found in the Appendix by index number.

## References

[CR1] Adam, B. D. (2006). Relationship innovation in male couples. *Sexualities*, *9*(1), 5–26. 10.1177/1363460706060685. 10.1177/1363460706060685

[CR2] Adam, B. D. (2016). Neoliberalism, masculinity, and HIV risk. *Sexuality Research & Social Policy*, *13*, 321–329. 10.1007/s13178-016-0232-2. 10.1007/s13178-016-0232-2

[CR3] Adam, B. D. (2020). Political economy, sexuality, and intimacy. In M. Bosia, S. M. McEvoy, & M. Rahman (Eds.), *The Oxford handbook of global LGBT and sexual diversity politics* (pp. 31–42). Oxford University Press.

[CR4] Bauman, Z. (2003). *Liquid Love*. Polity.

[CR5] Beck, U., & Beck-Gernsheim, E. (1995). *The normal Chaos of Love*. Polity.

[CR6] Blackwell, C., Birnholtz, J., & Abbott, C. (2014). Seeing and being seen. *new Media & Society*, *17*(7), 1117–1136. 10.1177/1461444814521595. 10.1177/1461444814521595

[CR7] Brennan, D., Kesler, M., Lachowsky, N. J., Davies, A., Georgevski, G., Adam, B. D., & Griffiths, D. (2022). Sociodemographic and psychological predictors of seeking health information online among GB2M in Ontario. *International Journal of Sexual Health*, *34*(2), 337–350. 10.1080/19317611.2021.2000087. 38596527 10.1080/19317611.2021.2000087 PMC10903557

[CR8] Choi, S., & Bauermeister, J. (2022). A latent profile analysis of online dating patterns among single young men who have sex with men. *AIDS and Behavior*, *26*, 1279–1288. 10.1007/s10461-021-03485-5. 34609630 10.1007/s10461-021-03485-5 PMC8940610

[CR9] Filice, E., Parry, D., & Johnson, C. (2021). Traditions in (re)negotiation. *Sexuality & Culture*, *25*, 189–216. 10.1007/s12119-020-09765-x. 10.1007/s12119-020-09765-x

[CR10] Ghaziani, A. (2014). *There goes the Gayborhood?* Princeton University Press.

[CR11] Giano, Z. (2019). The influence of online experiences. *Journal of Homosexuality*, *68*(5), 872–886. 10.1080/00918369.2019.1667159. 31532331 10.1080/00918369.2019.1667159

[CR12] Giddens, A. (1992). *The Transformation of Intimacy*. Stanford University Press.

[CR13] Goedel, W., & Duncan, D. (2015). Geosocial-networking app usage patterns of gay, bisexual, and other men who have sex with men. *JMIR Public Health and Surveillance*, *1*(1), e4. 10.2196/publichealth.4353. 27227127 10.2196/publichealth.4353 PMC4869243

[CR14] Green, A. (Ed.). (2014). *Sexual fields*. University of Chicago Press.

[CR15] Hammack, P., Frost, D., Meyer, I., & Pletta, D. (2018). Gay men’s health and identity. *Archives of Sexual Behavior*, *47*, 59–74. 10.1007/s10508-017-0990-9. 28585157 10.1007/s10508-017-0990-9 PMC5903851

[CR16] Hammack, P., Grecco, B., Wilson, B., & Meyer, I. (2021). White, tall, top, masculine, muscular. *Archives of Sexual Behavior*. 10.1007/s10508-021-02144-z. 10.1007/s10508-021-02144-z PMC9293832 34820783

[CR17] Illouz, E. (2007). *Cold intimacies*. Polity.

[CR18] Jepsen, L., & Jepsen, C. (2002). An empirical analysis of the matching patterns of same-sex and opposite-sex couples. *Demography*, *39*(3), 435–453. 10.1353/dem.2002.0027. 12205751 10.1353/dem.2002.0027

[CR19] Lauckner, C., Truszczynski, N., Lambert, D., Kottamasu, V., Meherally, S., Schipani-McLaughlin, A. M., & Hansen, N. (2019). Catfishing, cyberbullying, and coercion. *Journal of Gay & Lesbian Mental Health*, *23*(3), 289–306. 10.1080/19359705.2019.1587729. 10.1080/19359705.2019.1587729

[CR20] Lennes, K. (2021). Queer (post-)migration experiences. *Sexualities*, *24*(8), –1003. 10.1177/1363460720944591.

[CR21] Licoppe, C. (2020). Liquidity and attachment in the mobile hookup culture. *Journal of Cultural Economy*, *13*(1), 73–90. 10.1080/17530350.2019.1607530. 10.1080/17530350.2019.1607530

[CR22] Lundquist, J., & Lin, K. H. (2015). Is love (color) blind? *Social Forces*, *93*(4), 1423–1449. 10.1093/sf/sov008. 10.1093/sf/sov008

[CR23] Miles, S. (2017). Sex in the digital city. *Gender Place and Culture*, *24*(11), 1595–1610. 10.1080/0966369X.2017.1340874. 10.1080/0966369X.2017.1340874

[CR24] Miles, S. (2018). Still getting it on online. *Geography Compass*, *12*, e12407. 10.1111/gec3.12407. 10.1111/gec3.12407

[CR25] Mowlabocus, S. (2021). *Interrogating Homonormativity*. Palgrave Macmillan.

[CR26] Paska, I. (2020). Fast choices and emancipatory spaces. *In Media Res*, *9*(16), 2545–2557. 10.46640/imr.9.16.6. 10.46640/imr.9.16.6

[CR27] Regan, H. (2021). Fields, features, and filters. *Sexualities*. 10.1177/13634607211056878. 10.1177/13634607211056878

[CR28] Smith, J., Flowers, P., & Larkin, M. (2009). *Interpretative phenomenological analysis*. Sage.

[CR29] Stacey, J. (1990). *Brave new families*. Basic Books.

[CR30] Weeks, J. (2007). *The World we have Won*. Routledge.

[CR31] Weeks, J., Heathy, B., & Donovan, C. (2001). *Same sex intimacies*. Routledge.

[CR32] Weston, K. (1991). *Families we choose*. Columbia University.

[CR33] Woltersdorff, V. (2011). Paradoxes of precarious sexualities. *Cultural Studies*, *25*(2), 164–182. 10.1080/09502386.2011.535984. 10.1080/09502386.2011.535984

[CR34] Wu, S., & Ward, J. (2018). The mediation of gay men’s lives. *Sociology Compass*, *12*, e12560. 10.1111/soc4.12560. 10.1111/soc4.12560

[CR35] Wu, S., & Ward, J. (2020). Looking for interesting people. *Mobile Media & Communication*, *8*(3), 342–359. 10.1177/2050157919888558. 10.1177/2050157919888558

